# Factors predicting resolution of left ventricular thrombus in different time windows after myocardial infarction

**DOI:** 10.1186/s12872-024-03898-9

**Published:** 2024-05-29

**Authors:** Zhen Lu, Bingxue Song, Xin Liu, Ning Zhang, Fuhai Li, Fengqiang Xu, Zhexun Lian, Junjie Guo

**Affiliations:** 1 Department of Cardiology, The Affiliated Hospital of Qingdao University, Qingdao, Shandong China; 2 Qingdao University, Qingdao Medical College, Qingdao, China; 3 Qingdao Municipal Key Laboratory of Hypertension (Key Laboratory of Cardiovascular Medicine), Qingdao, Shandong China

**Keywords:** Myocardial infarction, Thrombosis, Embolism, Cardiovascular system, Left ventricular

## Abstract

**Background:**

Left ventricular thrombus (LVT) is a serious complication after myocardial infarction. However, due to its asymptomatic nature, early detection is challenging. We aimed to explore the differences in clinical correlates of LVT found in acute to subacute and chronic phases of myocardial infarction.

**Methods:**

We collected data from 153 patients who were diagnosed with LVT after myocardial infarction at the Affiliated Hospital of Qingdao University from January 2013 to December 2022. Baseline information, inflammatory markers, transthoracic echocardiograph (TTE) data and other clinical correlates were collected. Patients were categorized into acute to subacute phase group (< 30 days) and chronic phase group (30 days and after) according to the time at which echocardiograph was performed. The resolution of thrombus within 90 days is regarded as the primary endpoint event. We fitted logistic regression models to relating clinical correlates with phase-specific thrombus resolution.

**Results:**

For acute to subacute phase thrombus patients: C-reactive protein levels (OR: 0.95, 95% CI: 0.918–0.983, *p* = 0.003) were significantly associated with thrombus resolution. For chronic phase thrombus patients: anticoagulant treatment was associated with 5.717-fold odds of thrombus resolution (OR: 5.717, 95% CI: 1.543–21.18, *p* = 0.009).

**Conclusions:**

Higher levels of CRP were associated with lower likelihood of LVT resolution in acute phase myocardial infarction; Anticoagulant therapy is still needed for thrombus in the chronic stage of myocardial infarction.

**Supplementary Information:**

The online version contains supplementary material available at 10.1186/s12872-024-03898-9.

## Introduction

Left ventricular thrombus (LVT) refers to the presence of blood clots in the left ventricle. The formation of LVT after myocardial infarction is related to Virchow’s triad as the formation of other thrombus, including vascular intima injury [1], slow blood flow [2] and a hypercoagulable state [3]. Although the incidence of LVT decreased annually [4], it is still a nonnegligible complication after acute myocardial infarction (AMI) due to the serious consequences such as stroke and deformation of heart valves. Many previous studies have confirmed that anterior myocardial infarction, lower left ventricular ejection fraction (LVEF) [5, 6], and more severe inflammatory reaction [7] are related to the formation of LVT after AMI. Previous studies have shown that the most LVTs after AMI do not appear immediately after AMI, but occur after one week [8, 9]. The current timeframe available for diagnosis is often insufficient compared to the hospitalization time required for most patients after experiencing an AMI. This limitation hinders our ability to promptly detect and treat LVT cases, leading to an increase in embolic events and the formation of older, more challenging-to-resolve thrombus. The treatment of LVT after AMI is not fully clear, with previous studies yielding inconsistent conclusions regarding the use of anticoagulant drugs [10–14]. Given this, our aim is to investigate the differences in clinical correlates of phase-specific resolution of LVT after AMI.

## Methods

### Study design and population

This is a single-center retrospective study, authorized by the local institutional review board (Approval No.: QYFY WZLL 27,959). Diagnosis of AMI is made in patients who manifest a minimum of two of the following criteria: symptoms indicative of chest pain, elevated troponin (Tn) levels, and distinctive electrocardiogram (ECG) findings [15].

**Fig. 1:**
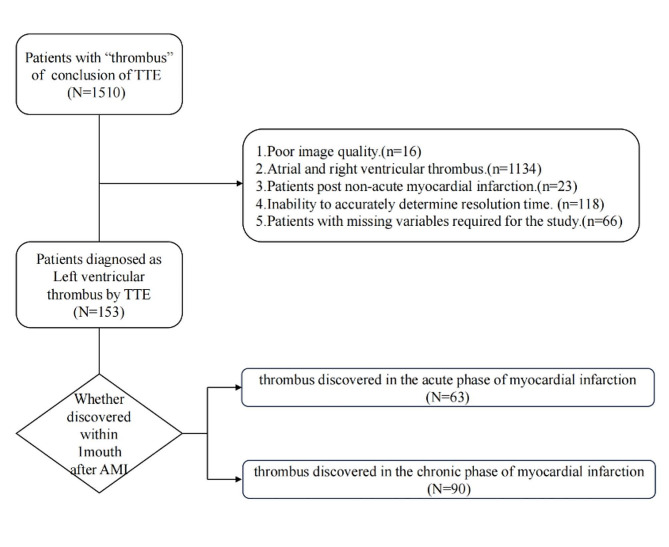
Sampling strategy

LVT detected through non-contrast transthoracic echocardiography (TTE) within the initial 30 days following AMI are assigned to the acute to subacute phase group (ALVT), whereas those detected beyond the 30 days after AMI are assigned to the chronic phase group (CLVT). A total of 1510 patients with documented occurrences of “thrombus” in their echocardiography reports were retrospectively assembled for this study from January 2013 to December 2022, drawing data from the Electronic Health Records (EHR) of the Qingdao University Affiliated Hospital. The following patients were excluded: (1) Poor image quality (*n* = 16); (2) Atrial and right ventricular thrombus (*n* = 1134); (3) Patients with non-acute myocardial infarction (*n* = 23); (4) Inability to accurately determine time to resolution(*n* = 118); (5) Patients with missing variables required for the study (*n* = 66). Ultimately, a cohort of 153 eligible patients presenting LVT was constituted, comprising 63 ALVT and 90 CLVT. They were dichotomized based on whether the thrombus resolved within 90 days post-detection, categorized into the early resolution group and the late resolution (including non-resolution) group. (Fig. 1) Their relevant baseline characteristics, biochemical and examination data, treatment, and outcomes were collected.

### Baseline and data collection

The resolution time of LVT was obtained through follow-up using echocardiography. LVEF, ventricular aneurysm, LVEDd, LVEDs and Fractional shortening was obtained by the initial echocardiographic data at the time of first LVT detection. Routine laboratory examinations when upon admission, including blood routine, renal function, coagulation function, and blood lipid analysis, were collected. Neutrophil-to-Lymphocyte Ratio (NLR) and Platelet-to-Lymphocyte Ratio (PLR) are obtained by dividing neutrophil count and platelet count by absolute lymphocyte count respectively, and lymphocyte-to- monocyte Ratio (LMR) is obtained by dividing lymphocyte count by Monocyte count. The use of beta-blockers, Angiotensin-converting enzyme inhibitor (ACEI), angiotensin receptor blockers (ARB), Angiotensin receptor-neprilysin inhibitors (ARNI), dual antiplatelet therapy, and anticoagulants was defined as the medication usage after the detection of LVT. Age, medical history, and other data are obtained from EHR. Patients with diagnosed LVT underwent regular echocardiograms every 3–6 months to monitor the resolution of the thrombus.

### Study outcomes

The end point events included early resolution, late resolution (including non-resolution) of LVT. Early resolution was defined as the disappearance of LVT detected in TTE within 90 days after the initial detection. If LVT persisted in TTE 90 days later, it was defined as late resolution.

### Statistical analysis

Continuous variables following a normal distribution were presented as mean ± standard deviation, while non-normally distributed continuous variables were represented by median and interquartile range. Analysis of variance (ANOVA) was used for continuously distributed variables that followed a normal distribution, the Mann-Whitney U test was used for those with non-normal distributions, and the chi-square test was used for categorical variables. We constructed a univariate logistic regression to detect impact of variables on thrombus resolution, including thrombus period, gender, age, smoking, diabetes, anticoagulation, LVEF, ventricular aneurysm, neutrophil count, lymphocyte ratio, CRP, triglycerides, low-density lipoprotein, and high-density lipoprotein. The above variables were then incorporated into the multivariate logistic regression model. Univariate logistic regression models and multivariate regression models were fitted separately for ALVT and CLVT. All analyses were performed using SPSS version 26.0 (SPSS Inc., Chicago, IL, USA). A two-sided significance level of 0.05 was used for all hypothesis tests.

## Results

### Comparison of LVT resolution and non-resolution at different stages

In this study, 153 patients with LVT were enrolled, with 63 (41.2%) having ALVT. Patients with ALVT have a higher probability of regression (*p* = 0.001), more use of PCI (*p* = 0.011) and anticoagulation (*p* = 0.001), Higher CRP (*p* = 0.001), higher fibrinogen (*p* = 0.003) and higher NT-proBNP (*p* = 0.012) than when CLVT patients were identified (Table 1). Among these patients, patients who had LVT resolution within 90 days compared with after 90 days had more anticoagulant medication (*p* = 0.047), triglyceride levels (*p* = 0.030), total bilirubin (*p* = 0.007), and indirect bilirubin (*p* = 0.022). Conversely, there were no statistically significant differences observed with respect to age, gender, medical history, medication history, or other laboratory indicators. (Table S1)

**Table 1:** AMI patients complicated by ALVT vs. CLVT

	All (*n* = 153)	ALVT (*n* = 63)	CLVT (*n* = 90)	*p*
**Resolution**, n (%)	77(50.3)	44(66.7)	33(36.7)	**< 0.001**
**Baseline characteristics and investigations**				
Age, mean ± SD	66.01 ± 11.06	64.43 ± 11.39	67.12 ± 10.74	0.139
Sex(man), n (%)	119(77.8)	47(74.6)	72(80)	0.429
BMI, mean ± SD	24.93 ± 3.6	25.18 ± 4.09	24.77 ± 3.24	0.505
Smoke, n (%)	76(49.7)	31(49.2)	45(50.0)	0.694
Drinking, n (%)	40(26.1)	16(25.4)	24(26.7)	0.687
Hypertension, n (%)	75(49.0)	28(44.4)	47(52.2)	0.344
Diabetes, n (%)	38(24.8)	19(30.2)	19(21.1)	0.202
**Revascularization**				
PCI, n (%)	76(49.7)	39(61.9)	37(41.1)	**0.011**
CABG, n (%)	16(10.4)	5(7.9)	11(12.2)	0.394
**Medications**				
Anticoagulants, n (%)	116(75.8)	62(98.4)	54(60)	**0.001**
LMWH, n (%)	38(24.8)	30(47.6)	8(8.9)	
Warfarin, n (%)	46(30.1)	22(34.9)	24(26.7)	
DOAC, n (%)	32(20.9)	10(6.5)	22(14.4)	
DAPT, n (%)	153(100)	63(100)	90(100)	1
Statins, n (%)	145(94.8)	60(95.2)	85(94.4)	0.822
ACEI, n (%)	17(11.1)	10(15.9)	7(7.8)	0.117
ARB, n (%)	12(7.8)	2(3.2)	10(11.1)	0.072
ARNI, n (%)	23(15.0)	10(15.9)	13(14.4)	0.808
β-blockers, n (%)	84(54.9)	40(63.5)	44(48.9)	0.074
**Echocardiographic findings**				
Ventricular aneurysm, n (%)	94(61.4)	40(63.5)	54(60.0)	0.416
LVEF, mean ± SD	43.9 ± 10.51	44.17 ± 9.08	43.71 ± 11.47	0.789
LVEDd, mean ± SD	5.34 ± 0.81	5.16 ± 0.67	5.47 ± 0.88	**0.018**
LVEDs, mean ± SD	4.07 ± 0.93	3.85 ± 0.77	4.25 ± 1.00	**0.021**
Fractional Shortening, mean ± SD	0.25 ± 0.08	0.25 ± 0.07	0.24 ± 0.08	0.318
**Inflammation biomarkers**				
Monocyte count, Median (IQR)	0.53(0.39–0.71)	0.67(0.47–0.96)	0.47(0.38–0.61)	**0.001**
Lymphocyte count, Median (IQR)	1.71(1.21–2.30)	1.7(1.1–2.45)	1.71(1.24–2.22)	0.915
Neutrophil count, Median (IQR)	4.69(3.49-8.0)	7.97(5.41–11.54)	3.68(3.01–4.54)	**0.001**
Lymphocyte ratio, Median (IQR)	23.7(14.7–30.7)	17.5(9.65–24.5)	27.35(21.85–34.18)	**0.001**
CRP, Median (IQR)	8.06(2.43–50.37)	41.87(14.1–83.9)	3.81(1.79–8.66)	**0.001**
NLR, Median (IQR)	2.79(1.88–5.13)	4.4(2.74–9.19)	2.29(1.64–3.06)	**0.001**
PLR, Median (IQR)	118.7(93.6-178.2)	134.5(99.8–243.0)	115.5(89.5-155.2)	0.066
LMR, Median (IQR)	3.08(2.0-4.28)	2.88(1.39–3.84)	3.42(2.47–4.55)	**0.004**
**Coagulation biomarkers**				
Fibrinogen, Median (IQR)	3.26(2.67–4.05)	3.58(2.76–4.81)	3.05(2.65–3.59)	**0.003**
D-dimer, Median (IQR)	450(270–1130)	510(270–1175)	430(280–1081)	0.906
PT, mean ± SD	12.1 ± 2.56	12.32 ± 2.54	11.95 ± 2.58	0.403
INR, mean ± SD	1.07 ± 0.23	1.07 ± 0.23	1.07 ± 0.24	0.869
**Lipid profile**				
TG, Median (IQR)	1.15(0.91–1.72)	1.18(0.94–1.95)	1.13(0.87–1.59)	0.116
LDL-C, Median (IQR)	2.53(1.98–3.06)	2.65(2.1–3.31)	2.52(1.93–2.94)	0.071
HDL-C, Median (IQR)	1.15(0.94–1.32)	1.21(0.97–1.32)	1.1(0.92–1.3)	0.35
**Heart failure**				
NT-proBNP, Median (IQR)	1436 (532–4205)	1963(1064–4615)	640(264.6–4012)	**0.012**
Tn, Median (IQR)	0.23(0.06–12.9)	17.36(4.53–59.9)	0.08(0.03–0.17)	**0.001**
**Other laboratory findings**				
Hb, mean ± SD	136.19 ± 23.59	135.87 ± 22.65	136.45 ± 24.46	0.887
Plt, mean ± SD	219.66 ± 78.13	238.72 ± 75.14	204.36 ± 77.59	**0.01**
MPV, Median (IQR)	10.15(9.53–10.80)	10.3(9.7–10.8)	10(9.3–11)	0.213
Total bilirubin, Median (IQR)	16.1(11.1–23.6)	17.65(11.4–24.4)	15.05(10.24–21.86)	0.257
direct bilirubin, Median (IQR)	4.87(3.2–7.43)	5.15(2.99–7.77)	4.77(3.46–7.19)	0.886
Indirect bilirubin, Median (IQR)	10.9(7.08–15.34)	12.05(7.52–15.48)	9.8(6.99–14.83)	0.22
Cr, mean ± SD	94.27 ± 43.92	92.93 ± 50.2	95.24 ± 35.01	0.756
UA, mean ± SD	374.6 ± 116.03	361.2 ± 103.16	383.59 ± 123.73	0.279

AMI - acute myocardial infarction; ALVT - thrombus discovered in the acute phase of myocardial infarction; CLVT - thrombus discovered in the chronic phase of myocardial infarction; BMI - Body Mass Index; PCI - Percutaneous Coronary Intervention; CABG - Coronary Artery Bypass Grafting; LMWH - Low Molecular Weight Heparin; DOAC - Direct oral anticoagulants; DAPT - Dual antiplatelet therapy; ACEI - Angiotensin-Converting Enzyme Inhibitor; ARB - Angiotensin II Receptor Blocker; ARNI - Angiotensin Receptor Neprilysin Inhibitor; LVEF - Left Ventricular Ejection Fraction; LVEDd - Left Ventricular End-Diastolic Dimension; LVEDs - Left Ventricular End-Systolic Dimension; CRP - C-reactive Protein; NLR - Neutrophil-to-Lymphocyte Ratio; PLR - Platelet-to-Lymphocyte Ratio; LMR - Lymphocyte-to-Monocyte Ratio; PT - Prothrombin Time; INR - International Normalized Ratio; TG - Triglycerides; LDL-C - Low-Density Lipoprotein Cholesterol; HDL-C - High-Density Lipoprotein Cholesterol; Tn – Troponin; Hb - Hemoglobin; Plt - Platelets; MPV - Mean Platelet Volume; Cr - Creatinine; UA - Uric Acid. Bold means a significant p-value of < 0.05

However, when patients are stratified according to the time of thrombus discovery, the statistical results differ from not discriminating time. Among the 63 cases of ALVT, 44 patients (69.8%) demonstrated thrombus resolution within 90 days. Variance analysis indicated no significant associations with age, gender, or medical history, while a significant relationship was observed with lower levels of inflammation (neutrophil count: 7.94 ± 3.94 vs. 10.12 ± 3.41, *p* = 0.049; lymphocyte ratio: 20.71 ± 11.92 vs. 13.37 ± 6.35, *p* = 0.020; C-reactive protein: 45.78 ± 43.6 vs. 107.54 ± 98.28, *p* = 0.015) (Table S2). Among the 90 cases of CLVT, only 33 cases (36.7%) exhibited thrombus resolution within 90 days. In the analysis of variance, male (*p* = 0.049), use of anticoagulant drugs (*p* = 0.001), and elevated creatinine levels (106.09 ± 52.1 vs. 88.56 ± 26.59, *p* = 0.045) were beneficial for thrombus regression. Additionally, younger age (64.58 ± 12.69 vs. 68.6 ± 9.23, *p* = 0.087) and lower triglyceride levels (1.43 ± 0.66 vs. 1.18 ± 0.57, *p* = 0.071) appeared to have some relevance to earlier thrombus resolution (Table S3).

### Predictors of LVT resolution in different discovery stage

In logistic regression that does not distinguish between thrombotic periods, we found that CRP and triglycerides are important factors affecting thrombus regression, and ALVT resolves more easily than CLVT (Table 2). Univariate logistic regression analyses were conducted separately for the ALVT and CLVT groups. In patients with ALVT, it was observed that neutrophil count (OR:0.86, 95% CI:0.75–1.003, *P* = 0.054), lymphocyte ratio (OR:1.081, 95%CI: 1.01–1.058, *p* = 0.026), and CRP (OR:0.986, 95%CI: 0.974–0.999, *p* = 0.035) were associated with thrombus resolution. (Table 3) Among CLVT patients, gender (OR:0.28, 95%CI: 0.074–1.054, *p* = 0.06), age (OR: 0.96, 95%CI: 0.92–1.006, *p* = 0.09), and the use of anticoagulants (OR: 5.76, 95%CI: 2.06–16.10, *p* = 0.001) were associated with thrombus resolution. However, after adjusting for covariates, only C-reactive protein remained as an independent predictor of thrombus resolution in ALVT (OR = 0.95; 95% CI 0.918–0.983; *p* = 0.003). In the multivariate logistic regression analysis, no statistically significant predictors for the resolution of CLVT were identified (Table 4).

**Table 2:** Univariate and multivariate Logistic regression of predictors of thrombus resolution in AMI patients with LVT

All patients	Univariate analysis		Multivariate analysis	
	OR (95% CI)	*p* value	OR (95% CI)	*p* value
ALVT	3.6(1.8–7.2)	0.001	86.4(5.8-1271.1)	**0.001**
Man	0.53(0.24–1.16)	0.11	0.22(0.038–1.25)	0.088
Age	0.98(0.95–1.01)	0.19	1.02(0.95–1.09)	0.053
Anticoagulants	4.41(0.91–21.50)	0.066	11.43(0.6-239.1)	0.087
Smoke	0.66(0.35–1.24)	0.19	0.327(0.077–1.39)	0.13
Diabetes	0.88 (0.42–1.84)	0.74	0.24(0.05–1.25)	0.09
LVEF	1.003(0.97–1.03)	0.83	0.95(0.88–1.026)	0.19
Ventricular aneurysm	1(0.52–1.92)	1	1.43(0.35–5.92)	0.62
Neutrophil count	1.05(0.96–1.16)	0.25	0.85(0.63–1.13)	0.25
Lymphocyte ratio	0.99(0.97–1.03)	0.89	0.99(0.91–1.08)	0.84
CRP	0.998(0.99–1.06)	0.64	0.98(0.97–0.99)	**0.041**
TG	1.73(1.05–2.85)	0.031	17.15(2.1-136.2)	**0.007**
LDL-C	1.24(0.85–1.80)	0.26	0.72(0.25–2.08)	0.54
HDL-C	1.49(0.48–4.71)	0.49	1.74(0.094–32.35)	0.71

**Abbreviations**: AMI - acute myocardial infarction; OR - odds radio; CI - confidence interval; LVT - left ventricular thrombus; ALVT - thrombus discovered in the acute phase of myocardial infarction; LVEF - Left Ventricular Ejection Fraction; CRP - C-reactive Protein; TG – Triglycerides; LDL-C - Low-Density Lipoprotein Cholesterol; HDL-C - High-Density Lipoprotein Cholesterol

Bold means a significant p-value of < 0.05

**Table 3:** Univariate and multivariate Logistic regression of predictors of thrombus resolution in AMI patients with ALVT

ALVT patients	Univariate analysis		Multivariate analysis	
	OR (95% CI)	*p* value	OR (95% CI)	*p* value
Man	0.63(0.19–2.11)	0.46	0.18(0.11–2.79)	0.22
Age	1.02(0.97–1.07)	0.42	1.006(0.90–1.12)	0.92
Anticoagulants	/^*^	/^*^	/^*^	/^*^
Smoke	0.66(0.22–1.97)	0.46	3.18(0.15–65.74)	0.45
Diabetes	1.10(0.34–3.52)	0.87	4.54(0.35–59.63)	0.25
LVEF	1.05(0.98–1.11)	0.14	1.05(0.92–1.2)	0.48
Ventricular aneurysm	0.71(0.24–2.14)	0.55	0.34(0.04–3.27)	0.35
Neutrophil count	0.86(0.75–1.003)	0.054	1.04(0.66–1.64)	0.88
Lymphocyte ratio	1.081(1.01–1.058)	0.026	1.19(0.94–1.49)	0.15
CRP	0.986(0.974–0.999)	0.035	0.95(0.92–0.98)	**0.003**
TG	1.27(0.67–2.42)	0.47	0.78(0.199–3.04)	0.72
LDL-C	1.32(0.68–2.58)	0.42	1.77(0.32–9.79)	0.51
HDL-C	8.52(0.73–98.98)	0.087	18.9(0.057–6246)	0.32

Bold means a significant p-value of < 0.05

**Abbreviations**: AMI - acute myocardial infarction; OR - odds radio; CI - confidence interval; LVT - left ventricular thrombus; ALVT - thrombus discovered in the acute phase of myocardial infarction; LVEF - Left Ventricular Ejection Fraction; CRP - C-reactive Protein; TG – Triglycerides; LDL-C - Low-Density Lipoprotein Cholesterol; HDL-C - High-Density Lipoprotein Cholesterol

* In the ALVT patients, the vast majority of them received anticoagulant treatment (62/63), so the relevant anticoagulant outcomes were not statistically analyzed

**Table 4:** Univariate and multivariate logistic regression of predictors of thrombus resolution in AMI patients with CLVT

CLVT patients	Univariate analysis		Multivariate analysis	
	OR (95% CI)	*p* value	OR (95% CI)	*p* value
Man	0.28(0.074–1.054)	0.06	0.27(0.047–1.59)	0.15
Age	0.96(0.92–1.006)	0.09	0.98(0.92–1.043)	0.54
Anticoagulants	5.76(2.06–16.10)	0.001	5.72(1.54–21.18)	**0.009**
Smoke	0.57(0.24–1.38)	0.22	0.79(0.22–2.91)	0.73
Diabetes	0.99(0.35–2.83)	0.99	0.73(0.17–3.15)	0.67
LVEF	0.98(0.95–1.023)	0.42	0.95(0.89–1.01)	0.1
Ventricular aneurysm	1.43(0.60–3.41)	0.42	0.92(0.28–3.10)	0.89
Neutrophil count	0.98(0.799–1.21)	0.88	0.94(0.67–1.31)	0.71
Lymphocyte ratio	1.007(0.961–1.056)	0.77	1.013(0.93–1.10)	0.76
CRP	1(0.98–1.018)	0.99	1.020(0.99–1.05)	0.13
TG	1.96(0.92–4.16)	0.080	2.25(0.83–6.10)	0.11
LDL-C	1.05(0.64–1.72)	0.84	0.55(0.23–1.29)	0.17
HDL-C	0.49(0.099–2.47)	0.39	0.66(0.05–8.69)	0.75

**Abbreviations**: AMI - acute myocardial infarction; OR - odds radio; CI - confidence interval; LVT - left ventricular thrombus; CLVT - thrombus discovered in the chronic phase of myocardial infarction; LVEF - Left Ventricular Ejection Fraction; CRP - C-reactive Protein; TG – Triglycerides; LDL-C - Low-Density Lipoprotein Cholesterol; HDL-C - High-Density Lipoprotein Cholesterol. Bold means a significant p-value of < 0.05

Regarding treatment, the majority of ALVT patients (62/63) received dual antiplatelet therapy with anticoagulation following AMI. Among CLVT patients, 60% (54/90) underwent anticoagulant treatment, while 36 out of 90 did not receive any anticoagulant medication, and 54 out of 90 were treated with low molecular weight heparin, vitamin K antagonists (VKA), or direct oral anticoagulants (DOACs). A total of 44 (69.8%) ALVT patients achieved thrombus resolution, in contrast to 33 (36.7%) CLVT patients. However, it’s noteworthy that 50% of CLVT cases achieved thrombus resolution through anticoagulant therapy (27/54) (Table 1). After adjusting for covariates, logistic regression revealed that CLVT patients receiving anticoagulant therapy had a 5.71-fold higher incidence of early resolution compared to those without anticoagulant therapy (95% CI: 1.543–21.18; *p* = 0.009) (Table 4). There was no significant difference between Warfarin and DOAC in the resolution of acute and old LVT (ALVT: OR = 1.143, 95% CI 0.22–5.928, *p* = 0.874; CLVT: OR = 1.707, 95% CI 0.53–5.496, *p* = 0.37).

## Discussion

As a retrospective cohort study comparing LVT at different discovery times following AMI, this study explored the clinical correlates associated with thrombus resolution in both acute-to-subacute and chronic AMI phases. We observed that ALVT is more likely to achieve thrombus resolution compared to CLVT. Moreover, the correlates related to the resolution of left ventricular thrombus differed by AMI phases.Prior research has mainly focused on identifying the risk factors associated with thrombus formation following AMI. These studies have uncovered associations between thrombus formation and factors such as cardiac function, the severity of the inflammatory response, the presence of ventricular aneurysms [6, 7, 16, 17]. Furthermore, some studies have suggested that a heightened inflammatory response can hinder the resolution of LVT in non-PCI patients [18]. However, as the thrombotic and fibrinolytic propensity may varied by AMI phase [19, 20], few studies so far have focused on phase-specific thrombus resolution. Due to the fact that over 70% of LVT occurring 1 week after AMI [8, 9], this time window is shorter than the hospitalization time for most AMI patients, leading to less likelihood to promptly perform TTE to discover thrombus. Therefore, in this study, we addressed this point by considering the phase at which thrombus detection was performed.

The occurrence mechanism of LVT after AMI is similar to most thrombus formations and is associated with Virchow’s triad, which includes continuous activation of coagulation mechanisms [21], persistent inflammatory state [22], impaired ventricular wall motion [23], and endocardial injury [24]. In our study, we found varied results between ALVT and CLVT. ALVT resolves more easily than CLVT, and resolution of ALVT is associated with inflammation but is absent in CLVT, which may be related to the several reasons. First, ALVT is more prone to resolution compared to CLVT, possibly due to delayed detection and prolonged existence of CLVT. Second, it may also be related to partial organization of the thrombus and strong adherence of the thrombus to the ventricular wall. Finally, more aggressive use of antiplatelet and anticoagulant medications in the acute-to-subacute phase may also provide insights into our observations.

In recent years, research has found that inflammation is not limited to classic defensive pathophysiological processes, but also forms a complex regulatory network with coagulation, anticoagulation, and fibrinolysis systems [25]. The mutual promotion between the activation of the coagulation system and inflammatory response is an important cause of local thrombosis. Sia et al. showed in their study that NLR and PLR can predict the resolution of Left ventricular thrombus after non-PCI [18]. In our study, the lymphocyte ratio and neutrophil count seem to indicate similar results. In addition, Lechner’s study suggests a correlation between CRP and the prediction of LVT after AMI [7], and our study also found that CRP has significance in the resolution of thrombus.

At present, there are no clear guidelines for the treatment of old arterial thrombosis, but the use of anticoagulant therapy has become a consensus [26]. There is meta-analysis indicating that DOAC treatment for LVT is superior to warfarin [27]. Our study did not find a difference between the two, but this could also be due to the limited sample size. Our study once again confirms that anticoagulant therapy may be still effective for chronic phase ventricular thrombus. This suggests that for high-risk patients with thrombus, more frequent TTE examinations might be considered for the screening of thrombus earlier and initiate anticoagulant therapy. For patients with a significant elevation CRP after myocardial infarction, it is important to take echocardiographic examinations more frequently to prevent cannot resolve of the thrombus once it is formed. For patients with chronic stage thrombus, active anticoagulation therapy may be considered.

### Limitation

As a single center, retrospective study, this study has the following limitations. Firstly, the number of research subjects is relatively small, and the interval and frequency of echocardiography are different, making it difficult to accurately understand the specific time of thrombus resolution or occurrence. It is possible that the detection of LVT through TTE but not cardiac magnetic resonance (CMR) imaging for assessing LVT could result in the omission of certain cases. The lack of statistical data on anticoagulant medication dosage, time in therapeutic range (TTR), and duration of administration indicates the need for large-scale studies. Such studies would provide a more robust evaluation of the efficacy and optimal dosing strategies of anticoagulant drugs for the treatment of LVT.

## Conclusion

In general, ALVT is more likely to resolve compared to CLVT. Higher level of CRP is indicative of a lower likelihood of LVT resolution following AMI in ALVT but not CLVT. Additionally, anticoagulant therapy is still necessary for the management of LVT in chronic phase AMI.

### Electronic supplementary material

Below is the link to the electronic supplementary material.

Supplementary Material 1

## Data Availability

The datasets used and analysed during the current study available from the corresponding author on reasonable request.

## Abbreviations

LVT: Left Ventricular thrombus
TTE: Transthoracic echocardiograph
AMI: Acute myocardial infarction
ALVT: Acute phase of Left ventricular thrombus
CLVT: Chronic phase of Left ventricular thrombus
NLR: Neutrophil-to-Lymphocyte Ratio
PLR: Platelet-to-Lymphocyte Ratio
LMR: lymphocyte-to- monocyte Ratio
ACEI: Angiotensin-converting enzyme inhibitor
ARB: angiotensin receptor blockers
ARNI: Angiotensin receptor-neprilysin inhibitors
HER: Electronic Health Records
VKA: vitamin K antagonists
DOACs: direct oral anticoagulants
TTR: time in therapeutic range
