# Supplementary material for: Factors predicting resolution of left ventricular thrombus in different time windows after myocardial infarction
Source: BMC Cardiovasc Disord. 2024 May 29;24:278. doi: 10.1186/s12872-024-03898-9 (PMC11134760; doi:10.1186/s12872-024-03898-9)
Supplement: Supplementary file 1 — Supplementary Material 1 [file 12872_2024_3898_MOESM1_ESM.docx]

**Supplementary Data**

**Supplementary Table S1. AMI patients complicated by LVT with vs. without resolution**

|  | **All (n=153)** | **Resolution**  **Before 90 days**  **(n=77)** | **Resolution**  **After 90 days**  **(n=76)** | **P** |
| --- | --- | --- | --- | --- |
| **ALVT**, n (%) | 63(41.2) | 44(57.1) | 19(25.0) | **<0.001** |
| **Baseline characteristics and investigations** | |  |  |  |
| Age, mean ± SD | 66.01±11.06 | 64.87±11.92 | 67.17±10.05 | 0.20 |
| Sex(man), n (%) | 119(77.8) | 64(83.1) | 55(72.4) | 0.11 |
| BMI, mean ± SD | 24.93±3.60 | 25.22±3.75 | 24.62±3.44 | 0.32 |
| Smoke, n (%) | 76(49.7) | 42(54.5) | 34(44.7) | 0.26 |
| Drinking, n (%) | 40(26.1) | 18(23.4) | 22(28.9) | 0.46 |
| Hypertension, n (%) | 75(49) | 35(45.5) | 40(52.6) | 0.38 |
| Diabetes, n (%) | 38(24.8) | 20(25.9) | 18(23.7) | 0.74 |
| **Revascularization** | |  |  |  |
| PCI, n (%) | 76(49.7) | 39(50.6) | 37(48.9) | 0.81 |
| CABG, n (%) | 16(10.5) | 6(7.8) | 10(13.1) | 0.28 |
| **Medications** |  |  |  |  |
| Anticoagulants, n (%) | 116(75.8) | 70(90.9) | 46(60.5) | **0.047** |
| LMWH, n (%) | 38(24.8) | 25(32.5) | 13(17.1) |  |
| Warfarin, n (%) | 46(30.1) | 29(37.7) | 17(22.4) |  |
| DOAC, n (%) | 32(20.9) | 16(20.8) | 16(21.1) |  |
| DAPT, n (%) | 153(100) | 77(100) | 76(100) | 1 |
| Statins, n (%) | 145(94.8) | 76(98.7) | (90.8) | 0.54 |
| ACEI, n (%) | 17(11.1) | 10(13.0) | 7(9.2) | 0.46 |
| ARB, n (%) | 12(7.8) | 3(4.1) | 9(11.8) | 0.068 |
| ARNI, n (%) | 23(15) | 14(18.2) | 9(11.8) | 0.27 |
| β-blockers, n (%) | 84(54.9) | 46(59.7) | 38(50.0) | 0.23 |
| **Echocardiographic findings** | |  |  |  |
| Ventricular aneurysm, n (%) | 94(61.4) | 47(61.0) | 47(61.8) | 0.61 |
| LVEF, mean ± SD | 44±11 | 44±11 | 44±10 | 0.84 |
| LVEDd, mean ± SD | 5.34±0.8 | 5.34±0.92 | 5.35±0.68 | 0.93 |
| LVEDs, mean ± SD | 4.07±0.93 | 4.07±1.05 | 4.07±0.8 | 0.99 |
| Fractional Shortening, mean ± SD | 24.54±7.52 | 24.14±7.84 | 24.94±7.24 | 0.58 |
| LVEF (admission), mean ± SD | 42±10 | 42±12 | 42±11 | 0.88 |
| **Inflammation biomarkers** | |  |  |  |
| Monocyte count, Median (IQR) | 0.53(0.39-0.71) | 0.53(0.42-0.77) | 0.55(0.39-0.67) | 0.33 |
| Lymphocyte count, Median (IQR) | 1.71(1.21-2.30) | 1.71(1.24-2.24) | 1.67(1.17-2.33) | 0.57 |
| Neutrophil count, Median (IQR) | 4.69(3.49-8.0) | 5.24(3.62-8.24) | 4.09(3.35-7.35) | 0.17 |
| Lymphocyte ratio, Median (IQR) | 23.7(14.7-30.7) | 23.7(15.4-31.3) | 23.9(13.58-30.6) | 0.89 |
| CRP, Median (IQR) | 8.06(2.43-50.37) | 17.98(2.88-58.79) | 7.61(2.74-118.94) | 0.44 |
| NLR, Median (IQR) | 2.79(1.88-5.13) | 2.85(1.92-5.1) | 2.73(1.87-5.32) | 0.67 |
| PLR, Median (IQR) | 118.7(93.6-178.2) | 116.6(92.9-205.3) | 118.9(93.5-171.3) | 0.73 |
| LMR, Median (IQR) | 3.08(2.0-4.28) | 3.09(2.02-4.06) | 3.04(1.95-4.48) | 0.71 |
| **Coagulation biomarkers** |  |  |  |  |
| Fibrinogen, Median (IQR) | 3.26(2.67-4.05) | 3.33(2.7-4.29) | 3.15(2.65-3.81) | 0.33 |
| D-dimer, Median (IQR) | 450(270-1130) | 440(268-978) | 500(280-1270) | 0.31 |
| PT, mean ± SD | 12.10±2.56 | 12.06±2.54 | 12.14±2.60 | 0.84 |
| INR, mean ± SD | 1.07±0.23 | 1.09±0.28 | 1.04±0.17 | 0.29 |
| **Lipid profile** | |  |  |  |
| TG, Median (IQR) | 1.15(0.91-1.72) | 1.24(0.94-1.94) | 1.09(0.85-1.49) | **0.030** |
| LDL-C, Median (IQR) | 2.53(1.98-3.06) | 2.65(2.1-3.31) | 2.52(1.93-2.94) | 0.27 |
| HDL-C, Median (IQR) | 1.15(0.94-1.32) | 1.21(0.97-1.32) | 1.1(0.92-1.3) | 0.40 |
| **Heart failure** |  |  |  |  |
| NT-proBNP, Median (IQR) | 1436 (532 -4205) | 1479 (647-3254) | 1436(356-5106) | 0.81 |
| **Other laboratory findings** | |  |  |  |
| Hb, mean ± SD | 136.19±23.59 | 139.21±23.21 | 132.94±23.73 | 0.12 |
| Plt, mean ± SD | 219.66±78.13 | 226.24±75.18 | 212.58±81.16 | 0.31 |
| MPV, Median (IQR) | 10.15(9.53-10.80) | 10.3(9.7-11) | 10(9.35-10.8) | 0.31 |
| Total bilirubin, Median (IQR) | 16.1(11.1-23.6) | 18.4(12.6-26.1) | 14.7(9.8-19.6) | **0.007** |
| direct bilirubin, Median (IQR) | 4.87(3.2-7.43) | 5.3(3.52-8.43) | 4.25(3.13-7.18) | 0.11 |
| Indirect bilirubin, Median (IQR) | 10.9(7.08-15.34) | 11.88(8.84-15.96) | 9.19(6.85-13.85) | **0.022** |
| Cr, mean ± SD | 94.27±43.92 | 97.21±49.15 | 91.20±37.81 | 0.41 |
| UA, mean ± SD | 374.59±116.03 | 386.62±113.49 | 362.93±118.11 | 0.24 |

**Abbreviations:** AMI - acute myocardial infarction; ALVT - thrombus discovered in the acute phase of myocardial infarction; BMI - Body Mass Index; PCI - Percutaneous Coronary Intervention; CABG - Coronary Artery Bypass Grafting; LMWH - Low Molecular Weight Heparin; DOAC - Direct oral anticoagulants; DAPT - Dual antiplatelet therapy; ACEI - Angiotensin-Converting Enzyme Inhibitor; ARB - Angiotensin II Receptor Blocker; ARNI - Angiotensin Receptor Neprilysin Inhibitor; LVEF - Left Ventricular Ejection Fraction; LVEDd - Left Ventricular End-Diastolic Dimension; LVEDs - Left Ventricular End-Systolic Dimension; CRP - C-reactive Protein; NLR - Neutrophil-to-Lymphocyte Ratio; PLR - Platelet-to-Lymphocyte Ratio; LMR - Lymphocyte-to-Monocyte Ratio; PT - Prothrombin Time; INR - International Normalized Ratio; TG - Triglycerides; LDL-C - Low-Density Lipoprotein Cholesterol; HDL-C - High-Density Lipoprotein Cholesterol; Hb - Hemoglobin; Plt - Platelets; MPV - Mean Platelet Volume; Cr - Creatinine; UA - Uric Acid. Bold means a significant p-value of < 0.05.

**Supplementary Table S2. ALVT with vs. without resolution**

|  | **All (n=63)** | **Resolution**  **Before 90 days**  **(n=44)** | **Resolution**  **After 90 days**  **(n=19)** | | **P** |
| --- | --- | --- | --- | --- | --- |
| **Baseline characteristics and investigations** | |  |  | |  |
| Age, mean ± SD | 64.43±11.39 | 65.09±11.46 | 62.89±11.39 | | 0.487 |
| Sex(man), n (%) | 47(74.6) | 34(77.3) | 13(68.4) | | 0.459 |
| BMI, mean ± SD | 25.18±4.09 | 25.6±3.86 | 24.11±4.58 | | 0.219 |
| Smoke, n (%) | 16(25.4) | 11(0.25) | | 5(26.3) | 0.912 |
| Drinking, n (%) | 31(49.2) | 23(52.3) | 8(42.1) | | 0.459 |
| Hypertension, n (%) | 28(44.4) | 21(47.7) | 7(36.8) | | 0.425 |
| Diabetes, n (%) | 19(30.2) | 13(29.5) | 6(31.6) | | 0.872 |
| **Medications** |  |  |  | |  |
| Anticoagulants, n (%) | 62(98.4) | 44(100) | 18(94.7) | | 0.125 |
| **Inflammation biomarkers** | |  |  | |  |
| Monocyte count, mean ± SD | 0.77±0.42 | 0.76±0.44 | 0.79±0.36 | | 0.806 |
| Lymphocyte count, mean ± SD | 1.87±1.12 | 1.93±1.24 | 1.7±0.74 | | 0.477 |
| Neutrophil count, mean ± SD | 8.54±3.9 | 7.94±3.94 | 10.12±3.41 | | **0.049** |
| Lymphocyte ratio, mean ± SD | 18.66±11.11 | 20.71±11.92 | 13.37±6.35 | | **0.020** |
| CRP, M (IQR) | 41.87(14.07-83.94) | 37.71(7.01-66.04) | 98.17(20.01-176.77) | | **0.015** |
| NLR, M (IQR) | 4.40(2.74-9.19) | 3.34(2.39-8.8) | 6.03(3.77-9.48) | | 0.708 |
| PLR, M (IQR) | 134.48(99.8-242.9) | 127.3(100-235.2) | 160.49(88.35-250.9) | | 0.607 |
| **Lipid profile** |  |  |  | |  |
| TG, mean ± SD | 1.57±1.07 | 1.64±1.19 | 1.42±0.74 | | 0.468 |
| LDL-C, mean ± SD | 2.77±0.88 | 2.83±0.9 | 2.63±0.84 | | 0.422 |
| HDL-C, mean ± SD | 1.18±0.29 | 1.23±0.32 | 1.08±0.19 | | 0.085 |
| Cr, mean ± SD | 92.93±50.2 | 90.44±46.26 | 98.44±58.99 | | 0.569 |

BMI - Body Mass Index; CRP - C-reactive Protein; NLR - Neutrophil-to-Lymphocyte Ratio; PLR - Platelet-to-Lymphocyte Ratio; TG - Triglycerides; LDL-C - Low-Density Lipoprotein Cholesterol; HDL-C - High-Density Lipoprotein Cholesterol. Bold means a significant p-value of < 0.05.

**Supplementary Table S3. CLVT with vs. without resolution**

|  | **All (n=90)** | **Resolution before 90 days (n=33)** | **Resolution**  **after 90 days (n=57)** | **P** |
| --- | --- | --- | --- | --- |
| **Baseline characteristics and investigations** | |  |  |  |
| Age, mean ± SD | 67.12±10.74 | 64.58±12.69 | 68.6±9.23 | 0.087 |
| Sex(man), n (%) | 72(80.0) | 30(90.9) | 42(73.7) | **0.049** |
| BMI, mean ± SD | 24.77±3.24 | 24.74±3.59 | 24.78±3.05 | 0.956 |
| Smoke, n (%) | 24(26.7) | 7(21.2) | 17(29.8) | 0.301 |
| Drinking, n (%) | 45(50.0) | 19(57.6) | 26(45.6) | 0.193 |
| Hypertension, n (%) | 47(52.2) | 14(42.4) | 33(57.9) | 0.157 |
| Diabetes, n (%) | 19(21.1) | 7(21.2) | 12(21.1) | 0.986 |
| **Medications** |  |  |  |  |
| Anticoagulants, n (%) | 54(60) | 27(87.1) | 27(47.4) | **0.001** |
| **Inflammation biomarkers** | |  |  |  |
| Monocyte count, mean ± SD | 0.52±0.21 | 0.52±0.21 | 0.52±0.21 | 0.937 |
| Lymphocyte count, mean ± SD | 1.74±0.66 | 1.77±0.63 | 1.72±0.69 | 0.746 |
| Neutrophil count, mean ± SD | 4.22±2.31 | 4.17±2.42 | 4.25±2.26 | 0.885 |
| Lymphocyte ratio, mean ± SD | 27.64±10.04 | 28.09±8.92 | 27.4±10.68 | 0.776 |
| CRP, M (IQR) | 3.81(1.79-8.66) | 4.46(1.91-8.22) | 3.44(1.28-9.26) | 0.829 |
| NLR, M (IQR) | 2.29(1.64-3.06) | 2.25(1.56-2.8) | 2.38(1.65-3.72) | 0.442 |
| PLR, M (IQR) | 115.5(89.5-115.2) | 113.5(89-151.31) | 119.4(88.6-159.3) | 0.589 |
| **Lipid profile** |  |  |  |  |
| TG, mean ± SD | 1.26±0.61 | 1.43±0.66 | 1.18±0.57 | 0.071 |
| LDL-C, mean ± SD | 2.55±0.89 | 2.58±0.99 | 2.54±0.85 | 0.841 |
| HDL-C, mean ± SD | 1.14±0.29 | 1.1±0.22 | 1.16±0.32 | 0.396 |
| Cr, mean ± SD | 95.24±39.01 | 106.09±52.1 | 88.56±26.59 | **0.045** |

BMI - Body Mass Index; CRP - C-reactive Protein; NLR - Neutrophil-to-Lymphocyte Ratio; PLR - Platelet-to-Lymphocyte Ratio; TG - Triglycerides; LDL-C - Low-Density Lipoprotein Cholesterol; HDL-C - High-Density Lipoprotein Cholesterol. Bold means a significant p-value of < 0.05.
